# Endovascular Aneurysm Sac Embolization for Treatment of Ruptured Aneurysms in the Aortoiliac Segment Using N-Butyl-Cyanoacrylate

**DOI:** 10.3390/life13040919

**Published:** 2023-03-31

**Authors:** Karim Mostafa, Marie Schierenbeck, Jens Trentmann, Hannes Gottschalk, Julian Andersson, Julian Pfarr, Malte Sieren, Olav Jansen, Philipp J. Schäfer

**Affiliations:** 1 Department for Radiology and Neuroradiology, University Hospital Schleswig Holstein, Campus Kiel, 24105 Kiel, Germany; 2 Department of Radiology and Nuclear Medicine, University Hospital Schleswig-Holstein, Campus Lübeck, 23569 Lübeck, Germany; 3 Institute of Interventional Radiology, University Hospital Schleswig-Holstein, Campus Lübeck, 23569 Lübeck, Germany

**Keywords:** endovascular embolization of ruptured aneurysms, transarterial embolization, ruptured aneurysm treatment, N-butyl-cyanoacrylate embolization

## Abstract

**Background** Aneurysmal rupture in the aortoiliac segment is a severe, life-threatening condition. Nowadays, in addition to surgical treatment, the implantation of a covered stent graft constitutes a feasible, minimally invasive treatment option. A novel approach is the add-on of transarterial aneurysm sac embolization with N-butyl-cyanoacrylate (NBCA). Here, we report our experience of performing this add-on embolization procedure after endovascular aneurysm repair for complex ruptured aneurysms of the aortoiliac segment. **Material and Methods** We describe six patients (mean age of 75.2 years; all male) with ruptured aneurysms in the visceral aortic and aortoiliac segment in whom a high-volume transarterial aneurysm sac embolization was performed as an add-on therapy to the implantation of an aortic prosthesis. The aim of this add-on intervention was to achieve the definite embolization of the aneurysmal rupture site and to ensure the best possible aneurysmal sealing. We report the feasibility, technical success, and considerations of using NBCA as well as clinical and follow-up imaging results, given their availability. **Results** Technical success was achieved in all cases. Clinical success was achieved in four cases. No periprocedural complications or reinterventions were reported. The mean full procedure time was 107.8 min. The mean radiation dose was 12,966.1 cGy/cm^2^. A mean amount of 10.7 mL of NBCA mixed with lipiodol in a 1:3 to 1:5 ratio was used for all patients. Available follow-up imaging up to 36 months after the procedure showed no aneurysm progression or endoleaks. In two patients, the NBCA cast had almost fully dissolved over the course of follow-up. **Conclusions** Our study underscores the notion that aneurysm sac embolization using high volumes of NBCA with ethiodized oil as an embolic agent is a feasible and add-on treatment option for optimizing the exclusion of the aneurysm from patients with ruptured aneurysms in the aortoiliac segment.

## 1. Introduction

Aneurysmal disease of the infrarenal abdominal aorta and the aortoiliac segment represents a potentially life-threatening vascular condition if it remains unmonitored and untreated. Men are affected four times more often than women, with a prevalence ranging from 1.7 to 5% [1]. In addition to the best pharmacological management, the two main curative treatment options are surgical aortic repair and endovascular aneurysm repair by prosthesis implantation (EVAR), with the latter predominantly becoming the treatment of choice [2]. After EVAR, patients must adhere to a regulated follow-up examination schedule in which various imaging methods are used to assess the patency and correct function of the endograft, exclude endoleaks, and identify patients in need of a reintervention to prevent a secondary aneurysm growth or rupture [1,2,3].

Aortic or iliac artery aneurysm rupture requires immediate interventional or operative management. The location of the aneurysm rupture, presence of active bleeding, history of previous EVAR, and the patient’s clinical condition comprise essential factors for deciding whether further operative or interventional radiological treatment is needed. Recent large observational studies have shown reintervention rates due to aneurysm rupture after EVAR that range from 2.4% to 4.3% [4].

The goals of EVAR and re-EVAR in the setting of aneurysmal rupture include the complete coverage of the ruptured aneurysmal aortic segment and sealing the implanted prosthesis proximally and distally to the vessel wall to ensure that the aneurysm is completely excluded from systemic circulation and to stop any bleeding from the aneurysmal rupture site. Favorable anatomic conditions for successful treatment include, for example, no or only minimal vessel kinking, nonatherosclerotic vessel walls in the proximal and distal landing zone, and sufficient aneurysmal neck length. However, achieving optimal sealing may be difficult in patients with a complex anatomy and aneurysm geometry. Here, the use of additional embolic agents or coils introduced into the aneurysm sac is known to be a useful add-on interventional technique. Furthermore, embolic agents injected into the ruptured aneurysmal segment during EVAR allow for the direct targeting and sealing of the vessel wall defect.

In this study, we describe our experience performing transarterial aneurysm sac embolization and targeted rupture site embolization with N-butyl-cyanoacrylate (NBCA) combined with lipiodol as an add-on treatment for patients with a ruptured abdominal aortic or iliac artery. Furthermore, we summarize the available literature on the use of embolic agents in ruptured aneurysms and provide a comparison to the findings of our research. 

## 2. Material and Methods

Ethical approval for this retrospective analysis was waived by the local institutional review board. This analysis was conducted in accordance with the ethical standards of the 1964 declaration of Helsinki and its later amendments.

### 2.1. Study Design

All patients in whom a ruptured abdominal aortic or iliac artery aneurysm was minimally invasively repaired at our center between 2019 and 2022 and in whom additional aneurysm sac embolization and targeted rupture site embolization was performed were included in this study. Information on the technical success, intervention time, and radiation dosage of the full procedure, the amount and mixing ratio of applied embolic agent, the type and manufacturer of endograft, the catheters used for application of NBCA, and the occurrence of any periprocedural complications was retrospectively gathered. 

### 2.2. Interventional Technique

**Figure 1:**
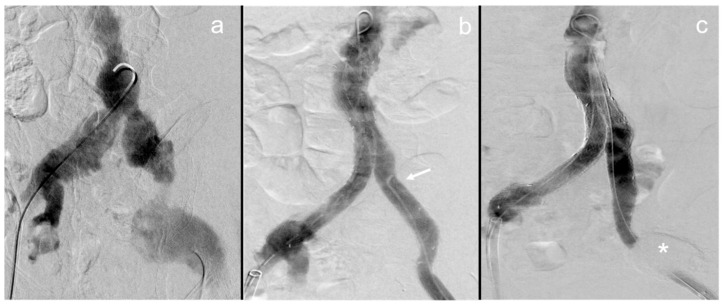
**Exemplary case of an aortobiiliac aneurysm with rupture of the left external iliac artery.** This patient presented with an aortobiiliac aneurysm with rupture of the left external iliac artery (**a**). After plug embolization of the left internal iliac artery, aneurysm repair was completed with standard EVAR while jailing a 5 French catheter inside the aneurysm at the rupture site (arrow, (**b**)). Finally, the rupture site was embolized with N-butyl-cyanoacrylate and lipiodol, which can be seen as a ball-shaped radiopacity on the left external iliac artery (star, (**c**)).

All procedures were performed percutaneously under local anesthesia with optional sedoanalgesia by one interventional radiologist (J.P.S) with more than 10 years of clinical experience in aortic interventions. Technical success was defined as the complete exclusion of the aneurysms from systemic perfusion and the cessation of any bleeding from the aneurysmal rupture site upon final angiograms. Immediately prior to prosthesis implantation, 5000–15,000 IE of heparin were administered, depending on patient weight and individual coagulation status. In this cohort, we used fenestrated aortic (E-nside, Jotec, Hechingen, Germany), aortobiiliac (Excluder, W.L. Gore & Associates, Newark, NJ, USA), and unilateral iliac stent grafts (Endurant and ETLW, Medtronic, Minneapolis, MN, USA) as well as aortic cuffs (Endurant IIs, Medtronic, Minneapolis, MN, USA) for endovascular treatment. Prior to graft implantation, a 4 or 5 French diagnostic catheter or sheath was placed in the aneurysm sac to be jailed by the implanted prosthesis. Ideally, this catheter was placed as near as possible to the rupture site, which could be determined by sac angiography in synopsis with previous CT imaging. Depending on the specific patient conditions, arteries arising from the aneurysm were first plug-embolized to eliminate retrograde aneurysm perfusion. Once the covered graft was successfully delivered, an estimation of the volume of the aneurysm sac from the catheter tip at the rupture site to the jailing point at the proximal or distal end of the endograft was performed to help define the optimal quantity of NBCA for embolization. A small amount of NBCA that would exit the rupture site into the retroperitoneal space, ensuring the definite sealing of the rupture site, was also taken into account. Finally, the aneurysm sac was filled with the previously determined mixture of NBCA and lipiodol under the removal of the catheter or sheath (Figure 1). Aneurysm sac embolization was performed under balloon-occlusion of the up- and downstream vessel segment if considered to be potentially threatened by non-target embolization. 

### 2.3. Clinical and Radiological Follow-Up

Follow-up imaging findings and clinical follow-up data were systematically gathered where available. Shock was defined as a systolic blood pressure < 80 mmHg or reduced consciousness. Coagulopathy was defined as an INR > 1.7 [1]. After the procedure, all eligible patients received follow-up imaging appointments for contrast-enhanced dual phase CT examinations when discharged from inpatient care or within 1 month after the procedure to enable risk stratification, as suggested by guidelines on follow-up after EVAR [1]. However, based on the complexity of the interventions and a history of aneurysmal rupture in all patients, yearly clinical follow-up appointments with point-of-care ultrasound imaging were scheduled at our center. If the complex situs could not be sufficiently evaluated, follow-up CT imaging was conducted. 

### 2.4. Statistics

Data were processed anonymously using Microsoft Excel and IBM SPSS. A descriptive statistical analysis of our study cohort was performed as indicated.

## 3. Results

### 3.1. Periprocedural Results

A total of six patients, all male, with complex ruptured aneurysms in the abdominal aorta and iliac arteries were retrospectively included in this study. Technical success was achieved in all cases. The median age of the patients was 76 years, ranging from 60 to 87 years. The median total intervention time, measured from the first to last fluoroscopic image, was 89 min, ranging from 28 to 269 min. The median radiation dose of the full procedure was 11,002.5 cGy/cm^2^, ranging from 2569 to 29,190 cGy/cm^2^. The median dose of heparin was 10,000 IE, ranging from 5000 to 15,000 IE. Two patients received unilateral graft implantation. One patient received a conventional aortobiiliac prosthesis implantation. One patient had a branched unilateral iliac prosthesis implanted. One patient received an aortic cuff, and one received a fenestrated aortic graft. For aneurysm sac embolization, a mean of 10.75 ± 2.4 mL of embolic agent was used, consisting of a mean of 2.33 mL (1.5–3 mL) NBCA combined with 8.4 mL (4.5–10 mL) of lipiodol. 

### 3.2. Case Descriptions

Since this procedure is complex and must be trimmed to specific patient and aneurysm characteristics, the following section briefly describes each patient’s case.

Patients no. 1 and 2 were both diagnosed with a ruptured left-sided internal iliac artery aneurysm and a large retroperitoneal hematoma. The aneurysms were located in the proximal internal iliac artery and measured approximately 6 × 6 × 6 cm in both patients. Firstly, the left internal iliac artery distal of the aneurysm was plug-embolized (Amplatzer Plug II, Abbott Laboratories, Chicago, IL, USA) in both patients. A 6 French sheath (Destination, Terumo, Tokyo, Japan) was placed inside the aneurysm sacs before remodeling the common and external iliac artery with a covered stent graft (Endurant and ETLW, Medtronic, Minneapolis, MN, USA). After successfully deploying the stent graft, the aneurysm sacs were filled with 6 and 11 mL of NBCA—lipiodol with a NBCA content of 25% and 27%—via a 5 French diagnostic catheter, which was introduced over the previously jailed 6 French sheath. 

The third patient presented with an infrarenal aortobiiliac aneurysm with rupture of the left external iliac artery and an extensive retroperitoneal hematoma. Conventional EVAR (Excluder, W.L. Gore & Associates, Newark, NJ, USA) was performed with prior plug-embolization of the left internal iliac artery and the jailing of a 4 French catheter (BER II, Cordis Corporation, Hialeah, FL, USA) at the rupture site of the left external iliac artery. Finally, the aneurysm was embolized with 13 mL NBCA—lipiodol with a NBCA content of 23%. 

A thoracoabdominal aneurysm with intra-abdominal rupture was found in the fourth patient. The aneurysm involved the distal thoracic aorta and the visceral aortic segment and measured 5.8 × 8.3 × 11.4 cm. Firstly, a branched stent graft (E-nside Design, Jotec, Hechingen, Germany) was delivered and adequately placed in the abdominal aorta while jailing a 5 French catheter (BER II, Cordis Corporation, Hialeah, FL, USA) in the aneurysm sac. Remodeling of the superior mesenteric and left renal artery was completed with covered stent grafts (Viabahn, W.L. Gore & Associates, Newark, NJ, USA). Regarding the specific anatomic conditions present in this case, namely, a shrunken right kidney, a renal artery that could not be probed and an occluded ostium of the celiac trunk, the remaining branches of the endograft were intentionally plug-embolized. Finally, the right renal artery and the aneurysm sac were embolized over the previously jailed catheter with 12 mL NBCA—lipiodol with a NBCA content of 16%. The procedure was completed with conventional TEVAR.

The fifth patient had history of previous aortobiiliac EVAR and presented with a re-rupture of the same aneurysm. The aneurysm ruptured due to a type Ia endoleak. Under intermittent balloon occlusion of the distal thoracic aorta, a 5 French catheter was placed transbrachially (BER II, Cordis, Hialeah, FL, USA) in the aneurysm. Secondly, an aortic cuff (Endurant IIs, Medtronic, Minneapolis, MN, USA) was introduced transinguinally to establish proximal sealing, jailing the catheter in the aneurysm sac. Subsequently, the aneurysm sac and rupture site were embolized under removal of the 5 French catheter with 10.5 mL of NBCA—lipiodol with a NBCA content of 23%. Patient no. 6 was diagnosed with a ruptured infrarenal aortic aneurysm measuring 7.4 × 7.0 × 9.3 cm. Four years previously, a standard EVAR procedure had been performed for the same aneurysm. A type 1b endoleak was identified as cause of the rupture, with the rupture site localized at the left posterolateral wall of the aorta. It had developed due to the cranial migration of the prosthesis, with the left iliac branch slipping out of the iliac artery. Firstly, the iliac branches were extended to re-establish patency to the pelvic axis. Secondly, the left internal iliac artery was remodeled with a fenestrated graft (E-liac, Jotec, Hechingen, Germany). Lastly, the rupture site was embolized over a previously jailed diagnostic catheter in the aneurysm sac using 12 mL of NBCA—lipiodol with a NBCA content of 16%.

### 3.3. Baseline and Postinterventional Clinical Parameters

Clinical pre- and postinterventional parameters and the duration of stay are listed in Table 1.

As past medical history, four patients suffered from arterial hypertension, and one had a previous history of coronary disease that was treated with stenting. Two patients had a history of previous EVAR. Three patients were referred from external hospitals.

Prior to the interventions, hemodynamic shock was present in one case (Patient no. 5). Patient no. 3 presented with acute-on-chronic kidney failure, with an estimated glomerular filtration rate (eGFR) of 16 mL/min/m2. Regarding hemoglobin levels, the lowest reported hemoglobin was 7.2 g/dL in patient no. 5 prior to the intervention, with the others ranging from 10.9 to 15.2 g/dL. Patient no. 6 showed signs of coagulopathy, with an INR of 3.19. No cases of cardiopulmonary resuscitation were noted.

After the intervention, two patients (no. 3 and 5) were in need of pharmacological circulatory support, and one patient (no. 3) was intubated due to progressive cardiac and respiratory failure. Both patients also received blood transfusions during and after the procedure (Table 1). 

Patients no. 2 and 6 were admitted to the normal ward given their hemodynamic stability. Patients no. 1, 3, 4 and 5 were admitted to the intensive care unit. Immediately after the procedure, patient no. 3, with a known history of coronary artery disease and despite technical procedural success, developed progressive cardiac failure and coagulopathy, resulting in lethal multi-organ failure within 12 h. Patient no. 5 developed severe lactic acidosis in the framework of hemodynamic shock within 48 h of the intervention. After careful assessment, no further therapy was initiated.

In the remaining patients, hemoglobin levels stabilized between 9.0 and 10.5 g/dL. One patient (no. 1) received a single blood transfusion eight hours after the intervention due to a slightly lowered, asymptomatic hemoglobin of 6.8 g/dL. Systolic blood pressures remained between 140 and 175 mmHg without the need for pharmacological circulatory support. No secondary renal failure or coagulopathy was reported. Until dismission from clinical care, no aneurysm related reinterventions were reported. One patient (no. 4) underwent hematoma evacuation two days after the intervention. 

### 3.4. Imaging Follow-Up Results

Follow-up imaging was conducted with two-phase, contrast-enhanced CT scans as the complex aortic conditions could not be precisely imaged otherwise. In concordance with our clinical standards, all patients received imaging examinations prior to being discharged from inpatient care or within one month after discharge. The mean follow-up period was 17.5 months, ranging from 3 to 35 months. 

Within 1 month after the procedure, patient no. 1 showed no signs of endoleakage or aneurysm progression and was reported to be symptom-free as of the latest clinical follow-up 5 months after the procedure. Further imaging follow-up was unavailable due to the patient living in a different state. 

**Figure 2:**
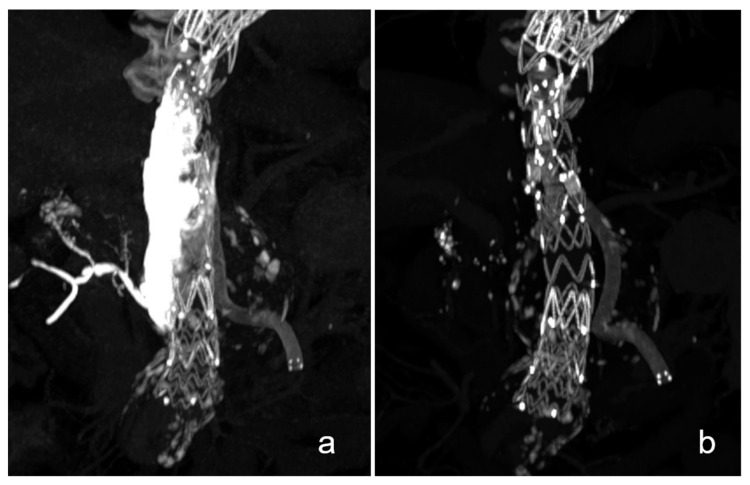
Dissolution of the NBCA cast in a patient after fenestrated endovascular aneurysm repair of the visceral aorta.

**Figure 3:**
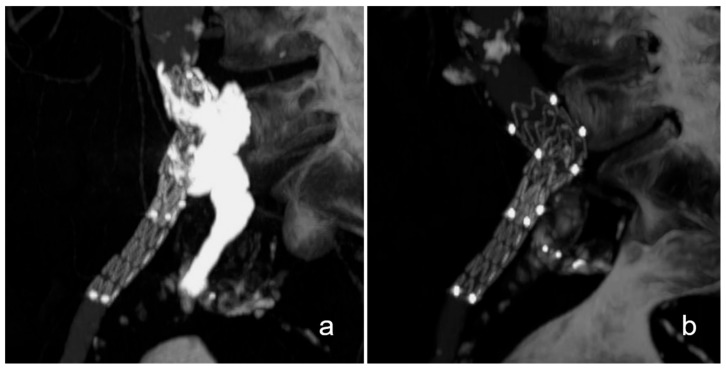
Dissolution of the NBCA cast 36 months after unilateral repair of the left iliac artery.

Patients no. 2 and 4 received continuous imaging follow-up up to 27 and 35 months after the procedure at which no signs of an endoleak were seen. Patient no. 4 showed a stable aneurysm diameter, while in patient 2, an aneurysm shrinkage of −1.9 cm was observed. In these patients, the instilled NBCA cast had almost completely dissolved over the course of follow-up up until 36 months after the procedure (Figure 2 and Figure 3). In both patients, only residual hyperdense fragments could be identified in the aneurysm sac on CT imaging. Patient no. 6 showed no occurrence of an endoleak, graft dysfunction or aneurysm diameter progression within 3 months after the embolization procedure. On the latest follow-up imaging, small retroperitoneal casts of NBCA could be seen, confirming small extravasations through the rupture site during the embolization procedure (Figure 3). 

In this patient, fenestrated endovascular aneurysm repair with add-on sac embolization and intentional right renal artery embolization with NBCA were conducted due to visceral aortic aneurysmal rupture. In the immediate postinterventional CT scan, the NBCA–lipiodol cast is seen inside the aneurysm sac and the right renal artery (a). In the follow-up imaging conducted after 26 months, the cast is almost completely dissolved, with only few remaining fragments in the right renal artery (b).

Aneurysmal rupture of the common left iliac artery in this patient was treated by implanting a covered stent graft with add-on NBCA embolization of the aneurysm rupture site. In the postinterventional scan, the NBCA cast is clearly visible (a). After 3 years, most of the cast has almost fully dissolved (b).

## 4. Discussion

In this research, we present six cases of acute and complex aortic or iliac artery aneurysm ruptures that were treated with NBCA—lipiodol embolization of the aneurysm sac and the targeted embolization of the aneurysm rupture site as an add-on therapy to endovascular aneurysm repair.

The main findings of this study are: (1) despite the complexity of the intervention, aneurysm sac embolization and targeted rupture site embolization were feasible and successful in all cases, and no instances of nontarget embolization were reported. (2) Available follow-up for up to 35 months after the procedure showed no occurrences of any type of endoleak, graft dysfunction or aneurysm diameter progression. (3) In two patients, the instilled NBCA cast had almost completely dissolved over the course of the follow-up period. 

### 4.1. N-Butyl-2-Cyanoacrylate

NBCA is a widespread embolic agent used for various embolization procedures. Cyanoacrylate itself is not radiopaque and needs to be administered as a two-component medium with lipiodol or tantalum powder. Once combined, it serves as a rapidly hardening embolic agent with various levels of viscosity that depend on the proportion of lipiodol [5]. In the setting of abdominal aortic aneurysm rupture, NBCA will be a reasonable embolic agent as its favorably high occlusive and adhesive properties are independent of a functioning coagulation system [6,7]. However, due to the known challenge in handling and necessary level of experience, embolization therapy with NBCA will be reserved for interventional specialists.

In our center, we normally use microcatheters (Progreat, Terumo, Tokyo, Japan) for transarterial delivery of NBCA. Before applying NBCA, we flush the microcatheters with a 40% glucose solution to avoid blockage of the catheter lumen. For aneurysm sac embolization, we used 4 to 5 French catheters (BER II, Cordis Corporation, Hialeah, FL, USA) or sheaths (Destination, Terumo, Tokyo, Japan) in a triaxial or coaxial approach with microcatheters. 

Data on long-term occlusion after NBCA embolization are still scarce, however, single histopathological reports have shown that the NBCA cast triggers a local inflammatory response [8,9]. 

### 4.2. Periprocedural Findings and General Considerations 

In the literature, different methods for aneurysm sac embolization, including coil and fibrin glue instillation via either percutaneous or endovascular access routes, have been described and investigated [10,11,12]. The earliest report of aneurysm sac embolization was published in 2005 by Zanchetta et al., in which a cohort of 64 patients with unruptured abdominal aortic aneurysms was investigated concerning the occurrence of type 2 endoleaks after sac embolization with fibrin glue [13]. Since then, a multitude of research studies have investigated both primary and secondary transarterial sac embolization for the prevention and treatment of type 2 endoleaks, using different embolic agents including coils, NBCA, Onyx, gelfoam and thrombin injection [13,14,15,16,17]. However, in the setting of aneurysmal rupture, primary transarterial embolization with targeting of the aneurysmal rupture site was not investigated until 2015. The first research in this field was conducted by Koike et al., who investigated three patients with ruptured abdominal aortic aneurysm. Of these patients, two underwent transarterial aneurysm sac and targeted rupture site embolization with NBCA—lipiodol [12]. 

Most recently, in 2020, research by Ohba et al. retrospectively analyzed aneurysm sac and rupture site embolization with NBCA for aortic aneurysm rupture in 22 patients [18]. They reported a mean intervention time of 108.5 min, which is in line with our mean intervention time of 107.8 min [18]. For elective standard percutaneous EVAR, a large meta-analysis reported mean intervention times to be approximately 108 min [19]. This underscores the notion that, though technically demanding, add-on sac and targeted rupture site embolization can be performed without significantly prolonging the intervention time, given the required level of expertise of the interventional radiologist. 

In our cohort, we noted a patient radiation exposure of 12,966.1 ± 8939.6 cGy/cm^2^. This is below the German national reference values for standard and branched aortic prosthesis implantation, which are set at 20,000 and 30,000 cGy/cm^2^ [20]. Naturally, radiation exposure in complex aortic procedures will be high. However, the additional radiation exposure generated by one sac angiography and approximately one minute of fluoroscopy during embolization will be negligible in the context of aneurysm repair (Video S1, Supplementary material). 

### 4.3. Clinical Outcomes

In this series, we present four successful cases of EVAR with embolization of the aneurysm sac and rupture site. In the short-term postinterventional follow-up, we report no instances of continuous bleeding, abdominal compartment syndrome or cardiopulmonary resuscitation. None of these patients needed circulatory support medication. Two patients were transferred to an ICU after the intervention simply for the purpose of continuous monitoring in the framework of aneurysm rupture, but these patients could be discharged within 3 days. Further, we report a shorter total length of hospital stay of 6.5 days in comparison to previous studies on this topic [18]. 

Despite technical success in all cases, we report early mortality in two patients. A meta-analysis in 2015 investigated factors associated with mortality in patients undergoing EVAR; in both patients, at least two of these factors were present, namely, ischemic heart disease, cardiac failure, hypertension and renal impairment [21]. Lastly, randomized trials have reported early mortality ranging from 18 to 35% for EVAR in ruptured aneurysms, and our results align with these statistics [22,23,24]. 

### 4.4. Targeted Rupture Site Embolization and Endoleak Management

Optimally, NBCA will serve as a direct seal at the rupture site. In the setting of aneurysm rupture, the goal of endovascular treatment must be to exclude the aneurysm from any systemic perfusion, and add-on sac embolization at the rupture site will reliably avoid the possibility of continuous bleeding after completion of the procedure. Any form of type 1 endoleak should be treated before concluding the primary endovascular therapy or as early as possible after being diagnosed, especially in the setting of aneurysmal rupture. Type 1 endoleaks pose a high risk for re-ruptures, potentially also in cases in which rupture site embolization has been conducted [25,26,27,28]. Management of these endoleaks will remain at the discretion of the interventionalist, with the simplest method being proximal or distal prosthesis extension [29]. For the treatment of a type 1 endoleak, embolization with NBCA will be reserved as a bail-out strategy if other techniques remain unsuccessful or are deemed unfeasible [10,30]. Here, Lu et al. presented excellent results for transarterial sac embolization, utilizing fibrin glue in the treatment of type 1a endoleaks among 35 patients who underwent EVAR. In these patients, previous attempts at addressing these endoleaks using other interventional methods had been unsuccessful [10].

Although they deliver less blood flow than type 1 endoleaks, type 2 endoleaks have also been reported to cause hemodynamically relevant bleeding out of the rupture site after standard EVAR, leaving the patient in need of an emergency secondary intervention [31]. Here, it is conclusive that if the rupture site is definitely embolized with NBCA, persistent bleeding due to an endoleak will be avoided. Further, re-rupture due to a type 2 endoleak is a very rare event, reported at 0.52% [28]. The endoleak can be addressed over the course of follow-up if deemed necessary. Notably, more than 50% of these endoleaks resolve spontaneously within one year. In theory, spatially distributed high volumes of embolic agent in the aneurysm sac, as are present in our cases, may affect development of a type 2 endoleak. Immediate type 2 endoleak avoidance in the setting of aneurysmal rupture is surely desirable; however, it is a goal secondary to the effective and complete sealing of the rupture site. In our cohort, no early or late type 2 endoleaks were diagnosed, even though the NBCA cast was seen to almost completely dissolve over the course of follow-up. Ohba et al., however, reported four type 2 endoleaks within 6 months in 22 patients, although aneurysm sac embolization with NBCA had been performed [18]. Lastly, the efficacy of higher volumes of embolic agents for avoidance of type 2 endoleaks remains to be fully investigated in larger scale trials. 

### 4.5. Considerations on Embolic Agent Volume and Embolization Technique 

**Figure 4:**
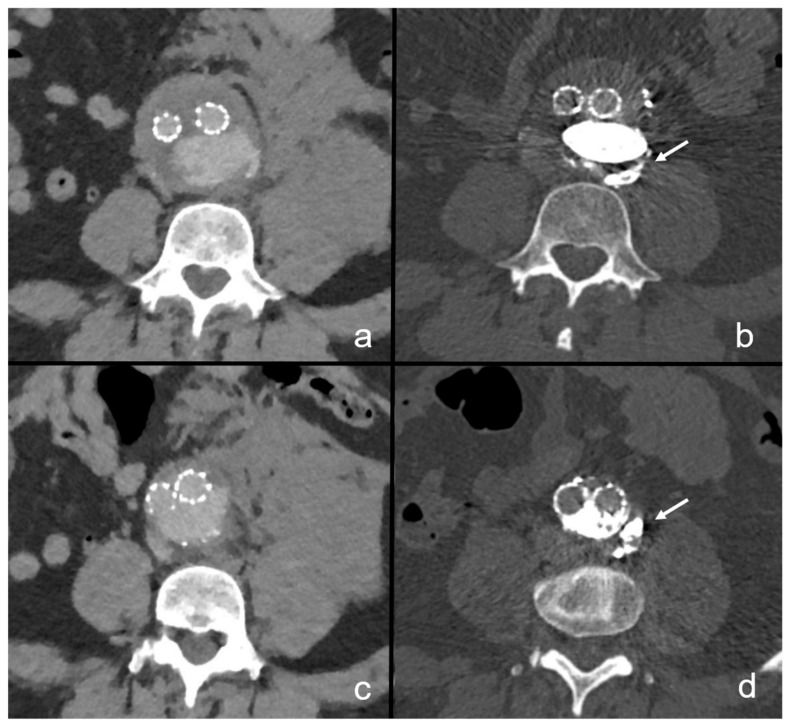
Retroperitoneal extravasation of NBCA immediately after embolization. In this patient, aneurysm rupture occurred due to a type 1b endoleak after previous EVAR. Images (**a**,**c**) present the ruptured situs with extensive left-sided retroperitoneal hematoma, suggesting location of the rupture site at the left aneurysm wall. This patient underwent endovascular prosthesis revision with sac embolization and targeted embolization of the rupture site with NBCA. Three months after the procedure, small residual retroperitoneal extravasations adjacent to the left aortic wall can be delineated, proving the rupture site to be occluded (**b**,**d**).

In our cohort, we report a mean total amount of embolic agent of 11 mL, consisting of a mean of 2.5 mL NBCA mixed with a mean of 8.5 mL lipiodol (Table 2). In the first study on this specific topic, Koike et al. reported the use of 1 mL NBCA/lipiodol mixed in a 50/50 ratio. Later, Ohba et al. reported the embolization of 4–5 mL NBCA/lipiodol with a NBCA content of 20–25% [12,18]. In comparison, we administered a higher mean amount of 10.75 mL in a similar mixing ratio, with NBCA contents ranging from 16 to 27%. It remains crucial to perform sac angiography to check for any major arteries arising from it that would be at risk of non-target embolization, especially large-caliber lumbar vessels supplying the spinal cord. Sac angiography can also aid in localization of the rupture site in a synopsis with previous CT imaging findings. The needed volume of embolic agent can be judged visually, as the amount and distribution of contrast dye allow a volumetric estimation of the non-thrombosed aneurysm lumen. When estimating the embolic volume, it is reasonable to add 0.5–1 mL to compensate for small extravasations of the embolic agent into the retroperitoneal space. Retroperitoneal extravasations are considered favorable, as this phenomenon proves the complete transversion of the ruptured segment of the aneurysm wall by NBCA, ensuring complete sealing (Figure 4) [18].

While embolizing the aneurysm sac, the timely and continuous retraction of the jailed catheter and sheath is crucial in order to avoid both becoming stuck to the NBCA cast, which may lead to catheter disruption. This presents a known and potentially severe complication when using NBCA as embolic agent [12,18,32]. Intermittent adherence of the NBCA cast to the delivering catheter will be unavoidable, and this effect may be mitigated with a higher-viscosity embolic agent composition with an increased proportion of lipiodol. In our study, the delivering catheter could be removed safely on all occasions. Here, we align with the research of Ohba et al., which considered concentrations of NBCA of 20–25% to be safe for this intervention [18].

Another method to facilitate the embolization procedure and minimize the risk of catheters sticking to the NBCA cast may be the use of microcatheters with detachable tips [33]. If using a triaxial approach with a sheath, catheter and microcatheter inside the aneurysm sac, the sheath can be used to strip the adhering NBCA cast off of the catheter tip and slightly push it back into the aneurysm sac while retracting the catheters. Lastly, however, high levels of expertise and experience in using NBCA will remain imperative to perform this procedure safely. 

### 4.6. Follow-Up Imaging and NBCA Dissolution

In two patients, continuous follow-up imaging up until 36 months after the procedure has shown that the NBCA cast progressively shrinks and dissolves (Figure 2 and Figure 3). The dissolution of NBCA was described in a report of eight patients after embolization of cerebral venous malformations by Rao et al. in 1989; however, no further reports or investigations on this topic are available to the present date [34]. Some reports have described different stages of allergic reactions and permanent vascular inflammation after NBCA embolization [35,36]. This is an interesting finding, especially when considering that neither of the two patients of our cohort showed any kind of endoleak or aneurysm progression. Furthermore, no deposition of hyperdense material in either the adjacent mesenteric and retroperitoneal tissue or distant to the embolization site was found on latest available follow-up imaging. In both of these patients, however, some small NBCA casts seem to have migrated within the aneurysm sac. These findings align with previous studies, which demonstrated an immunological reaction to NBCA. However, it remains unclear as to whether the dissolution of NBCA is of any relevance. This provides an interesting topic for further research. 

### 4.7. Study Limitations

While this study provides valuable insights into the treatment outcomes of rupture site and sac embolization with NBCA in patients with ruptured aneurysms in the aortoiliac segment, the small sample size, heterogenous aneurysms, and the single-center and retrospective design of the study limit the generalizability of the findings. As such, caution should be exercised when interpreting the results and applying them to patients with different aneurysm characteristics.

## 5. Conclusions

In this series, we show that aneurysm sac embolization with targeted rupture site embolization using higher volumes of NBCA than previously reported, aiming at maximized sealing of the aneurysmal lumen, is a feasible and safe add-on treatment option for patients with complex ruptured aneurysms in the aortoiliac segment. The clinical impact of our findings and of the almost full dissolution of the NBCA cast within 3 years remains to be elucidated.

## Figures and Tables

**Table 1:** Right side—Clinical parameters immediately prior to and five days after prosthesis implantation. Data are presented as median and range. Left side—Duration of hospital and ICU stay in days per patient.

	Prior to Intervention	Five Days Post Intervention	Duration of Hosptial Stay (ICU Stay)	Patient No.
Hemoglobin (g/dL)	13.1 (7.8–15.2)	9.65 (9–10.5)	6 (1)	1
Systolic blood pressure (mmgH)	120 (110–147)	150.5 (140–174)	5 (0)	2
Glomerular filtration rate (mL/min/1.73)	54 (16–84)	84 (47–93)	(1)	3
Creatinine (µmol/L)	119 (83–297)	83.75 (70–123)	8 (3)	4
INR	1.03 (0.96–3.19)	1.07 (1.01–1.67)	(2)	5
Blood transfusions (mL, range)	0	3 (1200, 300–2700)	7 (0)	6

**Table 2:** Summary of periprocedural and follow-up data.

Periprocedural Results										Follow-Up Results			
Patient No.	Sex	Age	Aneurysm Location	Graft Type	Intervention Time (min)	Radiation Dose (cGy/cm^2^)	Histoacryl (mL)	Lipiodol (mL)	Total Embolic Agent (mL)	Deaths	Latest Follow-Up (months)	Shrinkage at Latest Follow-Up (mm)	Reinterventions
1	Male	71	Left internal iliac artery	Unilateral	28	2569	1.5	4.5	6		5	0	0
2	Male	69	Left internal iliac artery	Unilateral	63	8996	3	8	11		35	−2	0
3	Male	87	Aortobiiliac	Bilateral	109	11,508	3	10	13	X			
4	Male	83	Thoracoabdominal aorta	Fenestrated	269	29,190	2	10	12		27	0	0
5	Male	81	Infrarenal aorta	Cuff	107	15,037	2.5	8	10.5	X			
6	Male	60	Infrarenal aorta	Bilateral	71	10,497	2	10	12		3	−3	0
Median		76 (60–87)			89 (28–269)	11,002.5 (2569–29,190)	2.25 (1.5–3) 9 (4.5–10)		11.5 (6–13)	20 (3–35) −1 (0–−3)			

X indicate that this patient died.

## Data Availability

The datasets generated and analysed during the current study are available form the corresponding author upon reasonable request.

## Supplementary Materials

The following supporting information can be downloaded at: https://www.mdpi.com/article/10.3390/life13040919/s1, Video S1: Transarterial embolization of the left internal iliac artery aneurysm lumen with NBCA.

## References

[1] Wanhainen A., Verzini F., Van Herzeele I., Allaire E., Bown M., Cohnert T., Dick F., van Herwaarden J., Karkos C., Koelemay M. (2019). Editor’s Choice—European Society for Vascular Surgery (ESVS) 2019 Clinical Practice Guidelines on the Management of Abdominal Aorto-iliac Artery Aneurysms. Eur. J. Vasc. Endovasc. Surg..

[2] National Institute for Health and Care Excellence (NICE) (2020). Abdominal Aortic Aneurysm: Diagnosis and Management [Nice Guideline 156]. https://www.nice.org.uk/guidance/ng156.

[3] Mostafa K., Pfarr J., Langguth P., Schäfer J.P., Trentmann J., Koktzoglou I., Edelman R.R., Neves F.B., Graessner J., Both M. (2022). Clinical Evaluation of Non-Contrast-Enhanced Radial Quiescent-Interval Slice-Selective (QISS) Magnetic Resonance Angiography in Comparison to Contrast-Enhanced Computed Tomography Angiography for the Evaluation of Endoleaks after Abdominal Endovascular Aneurysm Repair. J. Clin. Med..

[4] Goodney P., Mao J., Columbo J., Suckow B., Schermerhorn M., Malas M., Brooke B., Hoel A., Scali S., Arya S. (2022). Use of linked registry claims data for long term surveillance of devices after endovascular abdominal aortic aneurysm repair: Observational surveillance study. BMJ (Clin. Res. Ed.).

[5] Hill H., Chick J.F.B., Hage A., Srinivasa R.N. (2018). N-butyl cyanoacrylate embolotherapy: Techniques, complications, and management. Diagn. Interv. Radiol..

[6] Obata S., Kasai M., Kasai J., Seki K., Sekikawa Z., Torimoto I., Takebayashi S., Hirahara F., Aoki S. (2017). Emergent Uterine Arterial Embolization Using N-Butyl Cyanoacrylate in Postpartum Hemorrhage with Disseminated Intravascular Coagulation. Bio. Med. Res. Int..

[7] Kish J.W., Katz M.D., Marx M.V., Harrell D.S., Hanks S.E. (2004). N-butyl cyanoacrylate embolization for control of acute arterial hemorrhage. J. Vasc. Interv. Radiol..

[8] Quinn J.C., Mittal N., Baisre A., Cho E.-S., Sharer L.R., Gandhi C., Prestigiacomo C.J. (2011). Vascular inflammation with eosinophils after the use of n-butyl cyanoacrylate liquid embolic system. J. NeuroInterv. Surg..

[9] Brothers M.F., Kaufmann J.C., Fox A.J., Deveikis J.P. (1989). n-Butyl 2-cyanoacrylate—Substitute for IBCA in interventional neu-roradiology: Histopathologic and polymerization time studies. AJNR Am. J. Neuroradiol..

[10] Lu Q., Feng J., Yang Y., Nie B., Bao J., Zhao Z., Feng X., Pei Y., Yuan L., Mei Z. (2010). Treatment of type I endoleak after endovascular repair of infrarenal abdominal aortic aneurysm: Success of fibrin glue sac embolization. J. Endovasc. Ther..

[11] Fanelli F., Cannavale A., Chisci E., Citone M., Falcone G.M., Michelagnoli S., Miele V. (2021). Direct percutaneous embolization of aneurysm sac: A safe and effective procedure to treat post-EVAR type II endoleaks. Radiol. Med..

[12] Koike Y., Nishimura J., Hase S., Yamasaki M. (2015). Sac angiography and glue embolization in emergency endovascular aneurysm repair for ruptured abdominal aortic aneurysm. Cardiovasc. Interv. Radiol..

[13] Zanchetta M., Faresin F., Pedon L., Riggi M., Ronsivalle S. (2005). Fibrin glue aneurysm sac embolization at the time of endografting. J. Endovasc. Ther..

[14] Hongo N., Kiyosue H., Shuto R., Kamei N., Miyamoto S., Tanoue S., Mori H. (2014). Double coaxial microcatheter technique for transarterial aneurysm sac embolization of type II endoleaks after endovascular abdominal aortic repair. J. Vasc. Interv. Radiol..

[15] Müller-Wille R., Wohlgemuth W.A., Heiss P., Wiggermann P., Güntner O., Schreyer A.G., Hoffstetter P., Stroszczynski C., Zorger N. (2013). Transarterial embolization of type II endoleaks after Evar: The role of ethylene vinyl alcohol copolymer (Onyx). Cardiovasc. Interv. Radiol..

[16] Ronsivalle S., Faresin F., Franz F., Rettore C., Zanchetta M., Olivieri A. (2010). Aneurysm sac “thrombization” and stabilization in EVAR: A technique to reduce the risk of type II endoleak. J. Endovasc. Ther..

[17] Quinones-Baldrich W., Levin E.S., Lew W., Barleben A. (2014). Intraprocedural and postprocedural perigraft arterial sac embolization (PASE) for endoleak treatment. J. Vasc. Surg..

[18] Ohba S., Shimohira M., Hashizume T., Muto M., Ohta K., Sawada Y., Mizuno A., Nakai Y., Suda H., Shibamoto Y. (2020). Feasibility and Safety of Sac Embolization Using N-Butyl Cyanoacrylate in Emergency Endovascular Aneurysm Repair for Ruptured Abdominal Aortic Aneurysms or Isolated Iliac Artery Aneurysms. J. Endovasc. Ther..

[19] Wang Q., Wu J., Ma Y., Zhu Y., Song X., Xie S., Liang F., Gimzewska M., Li M., Yao L. (2023). Totally percutaneous versus surgical cut-down femoral artery access for elective bifurcated abdominal endovascular aneurysm repair. Cochrane Database Syst. Rev..

[20] Bundesamt für Strahlenschutz (2022). Bekanntmachung der Aktualisierten Diagnostischen Referenzwerte für Diagnostische und Interventionelle Röntgenanwendungen. https://www.bfs.de/SharedDocs/Downloads/BfS/DE/fachinfo/ion/drw-roentgen.pdf?__blob=publicationFile&v=9.

[21] Khashram M., Williman J.A., Hider P.N., Jones G.T., Roake J.A. (2016). Systematic Review and Meta-analysis of Factors Influencing Survival Following Abdominal Aortic Aneurysm Repair. Eur. J. Vasc. Endovasc. Surg..

[22] Amsterdam Acute Aneurysm Trial Collaborators (2013). Endovascular repair versus open repair of ruptured abdominal aortic aneurysms: A multicenter. J. Vasc. Surg..

[23] Powell J.T., Sweeting M.J., Thompson M.M., Ashleigh R., Bell R., Gomes M., Greenhalgh M., Grieve R., Heatley F., Hinchliffe R. (2014). Endovascular or open repair strategy for ruptured abdominal aortic aneurysm: 30 day outcomes from IMPROVE randomised trial. BMJ.

[24] Desgranges P., Kobeiter H., Katsahian S., Bouffi M., Gouny P., Favre J.-P., Alsac J., Sobocinski J., Julia P., Alimi Y. (2015). Editor’s Choice—ECAR (Endovasculaire ou Chirurgie dans les Anévrysmes aorto-iliaques Rompus): A French Randomized Controlled Trial of Endovascular Versus Open Surgical Repair of Ruptured Aorto-iliac Aneurysms. Eur. J. Vasc. Endovasc. Surg..

[25] Chaikof E.L., Dalman R.L., Eskandari M.K., Jackson B.M., Lee W.A., Mansour M.A., Mastracci T.M., Mell M., Murad M.H., Nguyen L.L. (2018). The Society for Vascular Surgery practice guidelines on the care of patients with an abdominal aortic aneurysm. J. Vasc. Surg..

[26] Buth J., Laheij R. (2000). Early complications and endoleaks after endovascular abdominal aortic aneurysm repair: Report of a multicenter study. J. Vasc. Surg..

[27] Harris P.L., Vallabhaneni S., Desgranges P., Becquemin J.-P., van Marrewijk C., Laheij R.J. (2000). Incidence and risk factors of late rupture, conversion, and death after endovascular repair of infrarenal aortic aneurysms: The EUROSTAR experience. European Collaborators on Stent/graft techniques for aortic aneurysm repair. J. Vasc. Surg..

[28] van Marrewijk C., Buth J., Harris P.L., Norgren L., Nevelsteen A., Wyatt M.G. (2002). Significance of endoleaks after endovascular repair of abdominal aortic aneurysms: The EUROSTAR experience. J. Vasc. Surg..

[29] Faries P.L., Cadot H., Agarwal G., Kent K., Hollier L.H., Marin M.L. (2003). Management of endoleak after endovascular aneurysm repair: Cuffs, coils, and conversion. J. Vasc. Surg..

[30] Chun J.-Y., Morgan R. (2013). Transcatheter embolisation of type 1 endoleaks after endovascular aortic aneurysm repair with onyx: When no other treatment option is feasible. Eur. J. Vasc. Endovasc. Surg..

[31] Ogawa Y., Nishimaki H., Chiba K., Ro D., Ono H., Sakurai Y., Fujiwara K., Murakami K., Hamaguchi S., Yagihashi K. (2015). Life-Saving Embolization in a Patient with Recurrent Shock Due to a Type II Endoleak after Endovascular Aortic Repair for a Ruptured Abdominal Aortic Aneurysm. Ann. Vasc. Dis..

[32] Ito M., Sonokawa T., Mishina H., Iizuka Y., Sato K. (1998). Disrupted and migrated microcatheter in the vertebrobasilar artery system in endovascular embolization of cerebellar AVM: Failure of endovascular and microneurosurgical retrieval. J. Clin. Neurosci..

[33] Paramasivam S., Altschul D., Ortega-Gutiarrez S., Fifi J., Berenstein A. (2015). N-butyl cyanoacrylate embolization using a detachable tip microcatheter: Initial experience. J. NeuroInterv. Surg..

[34] Rao V.R., Mandalam K.R., Gupta A.K., Kumar S., Joseph S. (1989). Dissolution of isobutyl 2-cyanoacrylate on long-term follow-up. AJNR Am. J. Neuroradiol..

[35] Sinha K.R., Duckwiler G., Rootman D.B. (2017). Urticarial reaction following endovascular embolization of an orbital arteriovenous malformation (AVM) with n-butyl cyanoacrylate (nBCA) glue. Interv. Neuroradiol..

[36] Rosen R.J., Contractor S. (2004). The use of cyanoacrylate adhesives in the management of congenital vascular malformations. Semin. Interv. Radiol..

