# Supplementary material for: Endovascular Aneurysm Sac Embolization for Treatment of Ruptured Aneurysms in the Aortoiliac Segment Using N-Butyl-Cyanoacrylate
Source: Life (Basel). 2023 Mar 31;13(4):919. doi: 10.3390/life13040919 (PMC10145858; doi:10.3390/life13040919)
Supplement: Supplementary file 1 [file life-13-00919-s001.zip › Table 1_Endovascular Aneurysm Embolization with NBCA.pdf]

Table S1. Summary of periprocedural and follow-up data.

Periprocedural Results											Follow-up Results		
Patient No.	Gender	Age	Aneurysm location	Type of implanted graft	Stent-graft	Intervention duration in minutes	Radiation dose in cGy/cm2	Histoacryl in ml	Lipiodol in ml	Volume of embolic agent in ml	Deaths	Latest available follow-up (months)	Reinterventions
1	Male	71	Left internal iliac artery	Unilateral	Endurant (Medtronic)	28	2569	1.5	4.5	6		5	0
2	Male	69	Left internal iliac artery	Unilateral	ETLW (Medtronic)	63	8996	3	8	11		35	0
3	Male	87	Aortobiiliac	Bilateral	Excluder (Gore Medical)	109	11508	3	10	13	X		
4	Male	83	Thoracoabdominal aorta	Fenestrated	Endurant (Medtronic) + cTAG (Gore Medical) + E-nside (Jotec)	269	29190	2	10	12		27	0
5	Male	81	Infrarenal aorta	Cuff	Endurant IIs (Medtronic)	107	15037	2.5	8	10.5	X		
6	Male	60	Infrarenal aorta	Bilateral	E-liac (Jotec) + Excluder (Medtronic)	71	10497	2	10	12		1	0
Mean		75.2 ± 10.2				107.8 ± 84.5	12966.1 ± 8939.6	2.33	8.42	10.75 ± 2.4			
